# Assembly of heteropoly acid into localized porous structures for *in situ* preparation of silver and polypyrrole nanoparticles†

**DOI:** 10.1039/c8ra07939k

**Published:** 2018-10-30

**Authors:** Jing Liang, Lei Yu, Jiangyong Zhang, Shixiong Zhao, Jiejing Zhang, Jianfeng Zhang

**Affiliations:** College of Life Science, Jilin Agricultural University Changchun 130118 China liangjing@jlau.edu.cn; Jilin Radion and TV University Changchun 130022 China; Dalian Institute of Chemical Physics, Chinese Academy of Science 116023 China

## Abstract

A simple and facile method to fabricate porous films which were locally patterned by heteropoly acid was developed in this study. The mixture of poly(methyl methacrylate) and stabilizer dichloromethane solution which contains heteropoly acid aqueous solution, prepared through shaking, was applied to fabricate a reversed microemulsion. After spreading and evaporating the solvent of microemulsion on a glass slide, an ordered honeycomb film was produced by incorporation of heteropoly acid in the cavities. The locally anchored heteropoly acid could be readily applied for the selective modification of the porous films through the *in situ* chemical reactions in the cavities with the additive agents. The silver nanoparticles were *in situ* prepared *via* the reduction of silver ions by reduced state H_3_PW_12_O_40_, and the polypyrrole spheres were locally obtained through the oxidative polymerization of pyrrole catalyzed by H_3_PMo_12_O_40_ in the cavities. Considering that water-soluble molecules and nanoparticles were universally suitable for the present strategy, the reported approach opened up an efficient way for patterning organically incompatible components on porous polymer films *via* the assembly of microemulsion droplet carriers to fabricate multi-functional hybrid surface structures.

## Abstract 2

An ordered heteropoly acid patterned porous structure is prepared which can be applied to the preparation of silver and polypyrrole nanoparticles.

## Graphical abstract

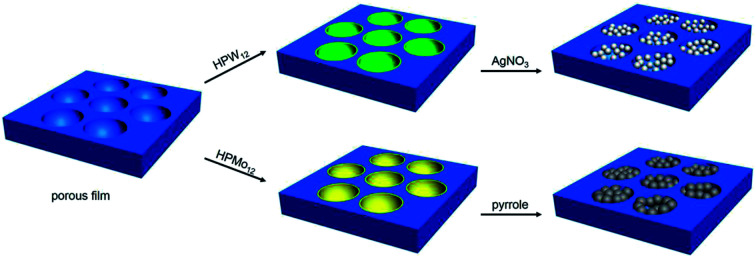

## Introduction

Owing to the significant applications of catalysis,^1^ photonics,^2^ membrane science,^3^ and so forth, porous materials have been widely developed. Among the various self-assembly methods used to prepare ordered surfaces, the breath figure technique, which uses water droplets as templates, has been widely investigated owing to its fast and easy operation features.^4,5^ As the porous films are composed of the polymer framework and cavities, their use can be applicable to frameworks and cavities. So far, the applications of the polymer framework have been widely developed, for example, acting as superhydrophobic surfaces,^6^ serving as the skeleton of cell adhesion and culture,^7,8^ use as photoelectronic devices,^9,10^ and so forth. Comparing with the vigorous progress of the framework, the usage of cavities is less reported, just acting as secondary templates^11^ and in protein recognition.^12^ Thus, how to develop the new functions of the cavities becomes very meaningful, which will bring new vitality to the field of porous films.

A series of additives such as titanium dioxide microparticles,^13^ proteins,^14^ carbon nanotubes,^15^ polystyrene microspheres,^16^ silica particles,^17^ graphenes^18^ and so on, have been assembled into the patterned cavities to present the functional features at the localized position, by using the breath figure method. However, the additives only standing in the localized sites, special features and applications have not been shown. To realize the application of cavities, the incorporation of functional materials into the cavities and selective modification of cavities are essential. However, for breath figure, the localized modification always needs multi-steps. Recently, a novel way to synthesize ordered porous structures using microemulsion droplets as template has been proposed.^19^ Water-phase additives locate at the inner walls of the cavities, which are suitable for further modification of the pore surface. This strategy maintains the advantages as those using in breath figure, while functional modifiers can be incorporated into the cavities in one step during the formation of porous structure.

**Scheme 1:**
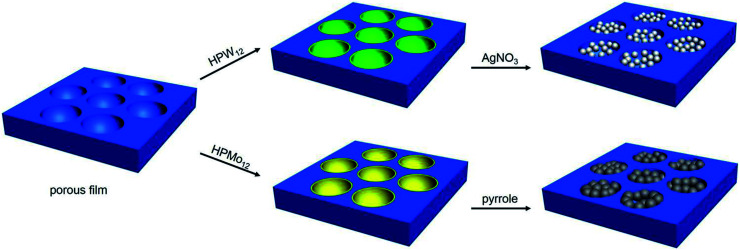
The schematic drawing of assembly of heteropoly acids and *in situ* reduction of silver ions and polymerization of pyrrole in the cavities by heteropoly acids.

Herein, honeycomb-patterned films, where the heteropoly acids were incorporated into the cavities and the polymer were spread on the framework, have been prepared through microemulsion method. This strategy exhibits some advantages. The heteropoly acids are locally assembled in the cavities on the polymer surface in one step. The excellent features of heteropoly acids patterned in the cavities are maintained through a series of treatment in the porous film preparation process. Interestingly, the heteropoly acids accumulated in the cavities of polymer surface can be used to *in situ* prepare silver and polypyrrole nanoparticles, which provide new functions for the cavities on the porous films (Scheme 1). It should be noted that the cavities were used as a micro-reactor at the first time, which brings the meaningful applications for the cavities, different from the additives incorporated into the cavities in the previous studies, revealing potential applications in microreaction, pattern recognition, cell culture and so forth.

## Experimental section

### Materials

H_3_PW_12_O_40_·*n*H_2_O (HPW_12_) and H_3_PMo_12_O_40_·*n*H_2_O (HPMo_12_) were purchased from Sinopham Chemical Reagent Co. Ltd. Poly(methyl methacrylate) (PMMA, *M*_w_: 349 kg mol^−1^), pyrrole, silver nitrate, PEO_20_-PPO_70_-PEO_20_ (P123), were the products of Sigma-Aldrich, Shanghai Kefeng Chemical Reagent Co. Ltd, Nanjing Chemical Reagent Co. Ltd, Anqiushi Luxing Chemical Co. Ltd. China, respectively.

### Preparation of heteropoly acids-incorporated porous film

The mixture of PMMA (6 mg mL^−1^) and P123 (0.5 mg mL^−1^) was prepared by simply adding them into a certain volume of dichloromethane. Heteropoly acids were dissolved in the distilled water (18.2 MΩ cm^−1^) at the concentration of 10 mg mL^−1^. For the preparation of microemulsion solution, the typical procedure was that to the dichloromethane solution of PMMA and P123 was added an aqueous HPMo_12_ solution under certain concentration, and the volume fraction of water was maintained at 5% unless it was mentioned. The mixture solution was shaken for 30 s under 25 °C to disperse the aqueous solution in organic phase and achieve a translucent gray microemulsion. Then, 20 μL of the microemulsion solution was cast onto a glass slide under the relative humidity of 30–40% at the temperature of 25 °C to achieve HPMo_12_/PMMA hybrid films. Following the similar procedures, HPW_12_/PMMA films were prepared.

### *In situ* polymerization of pyrrole

The pyrrole monomer with the concentration of 0.1 M was dissolved in a pH 4 aqueous solution, which was adjusted with diluted HCl. The HPMo_12_/PMMA film was dipped into the pyrrole solution for 40 min, washing with water three times and drying in air.

### *In situ* preparation of silver nanoparticles

For the preparation of silver nanoparticles, the HPW_12_/PMMA film was exposed at ultraviolet lamp within the distance of 15 cm for *ca.* 15 min. Then, the film was immediately dipped into the 0.1 M silver nitrate aqueous solution for 30 min, followed by washing with water three times and drying in air.

### Measurements

Scanning electron microscopy (SEM) images were collected on a JEOL JSM-6700F field emission scanning electron microscope. X-ray energy-dispersive spectroscopy (EDX) analysis was acquired on a JEOL FESEM 6700F electron microscope. X-ray photoelectron spectroscopic (XPS) analysis was performed on a VG Escalab MK-II spectrometer with an Al Kα (1486.5 eV) achromatic X-ray source. X-ray diffraction (XRD) pattern was collected on a Rigaku X-ray diffractometer (D/max rA, using CuK_α_ radiation at 1.542 Å). UV-Vis absorption spectra were obtained using a Varian Cary 50 UV-Vis spectrometer.

## Results and discussion

### Preparation and structural characterization of HPMo_12_/PMMA porous films

**Fig. 1:**
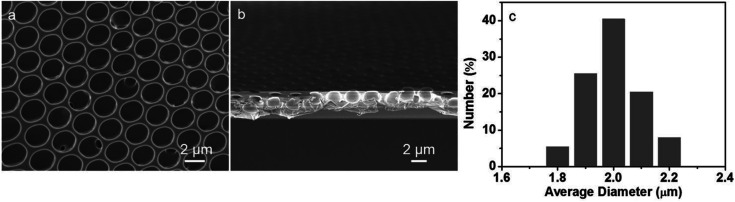
SEM images viewed from the (a) top surface and (b) cross section, and (c) histogram referring to the size distribution of cavities of the porous film prepared by casting the microemulsion solution on a glass slide.

According to the similar principle of breath figure for the preparation of ordered porous patterns on polymer surfaces, a different route for the preparation of heteropoly acids-patterned polymer films using microemulsion solution was applied. As a typical strategy, the microemulsion of PMMA dichloromethane solution bearing P123 were prepared by simply mixing with the aqueous solution of HPMo_12_ clusters and following a slight shaking, which makes the reversed microemulsion droplets disperse in the organic solution evenly. After spreading the sample solution on a solid substrate and the evaporation of solvent, the porous polymer film was obtained under the certain humidity. The obtained polymer film exhibits bright iridescent colors when viewed along the reflection light, indicating a periodic refractive index variation regarding the film thickness. The surface structure of the HPMo_12_/PMMA film was characterized through SEM measurement. The SEM image in Fig. 1a shows a highly ordered honeycomb-patterned film showing monodispersed hexagonal close-packed holes with a long range order forms in a large area without defect. Observation of the cross-section SEM, shows that the film exhibits a monolayer porous structure and the depth of cavities are approximately 2 μm, as seen in Fig. 1b. The histogram (Fig. 1c) illustrates the size distribution of the cavities with the diameter between 1.8 to 2.2 μm.

### Selective assembly of heteropoly acids into patterned cavities

**Fig. 2:**
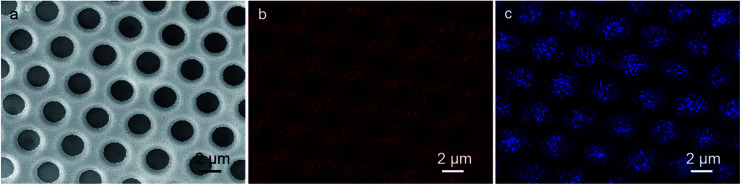
(a) SEM image and EDX analysis of HPMo_12_-containing film for (b) C in red, and (c) Mo in blue, respectively.

To realize the application of porous structures, the key step in the current research is to make the chemical difference of the cavities from the polymer films. The position of additive in the films is characterized through EDX analysis, which is an impactful method to determine the component distribution, due to the element difference between organic and inorganic components in the films. As Mo element only exists in HPMo_12_ cluster, and C element only exist in the polymers, the distribution difference of C and Mo elements can definitely figure out the location of the polymers and clusters in the porous film. By using the height SEM image (Fig. 2a) of the same patterned film as the reference, the C element marked in red (Fig. 2b) in the EDX image is found throughout the film with the isolated dark domains separating regularly in a hexagonal matrix distribution, indicating that the continuous phase is composed of the polymer PMMA. Because the inorganic component is supposed to cover on the inner wall of the cavities, it is reasonable that the C element cannot be detected, considering the shallow penetration of electron beam. In opposite to the distribution of the C element, as expected, the Mo element in blue color is observed fully locating in the isolated round domains that are regularly separated in a hexagonal matrix distribution, pointing out that the HPMo_12_ clusters distribute in the areas of cavities. The mutual compensation of the EDX pattern between C element and Mo element in Fig. 2b and c, and the consistency to the geometric pattern found in the SEM result also support the assignment of the chemical pattern. Thus, HPMo_12_ additive specifically modifies the inner walls of cavities (Scheme 1).

### Local polymerization of pyrrole monomer in the cavities

**Fig. 3:**
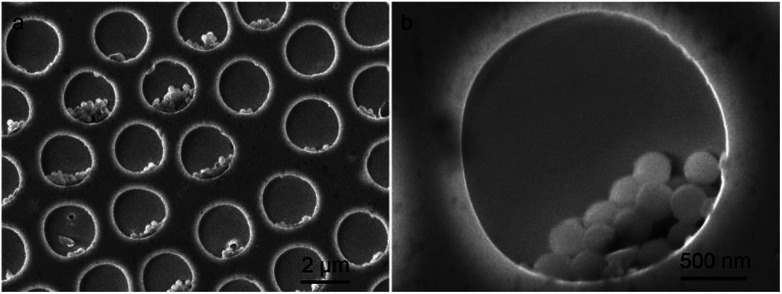
SEM images of (a) HPMo_12_/PMMA porous film containing polypyrrole spheres and (b) its amplification in one cavity, in which the film is prepared by dipping the hybrid porous film in 0.1 M pyrrole aqueous solution for *ca.* 40 min under pH 4.

The selective deposition of the water soluble heteropoly acids into the patterned cavities provides a facile route to functionalize the polymer surface locally. More interestingly, HPMo_12_, as a kind of strong oxidant and acid, was reported to be applicable for the oxidation polymerization of pyrrole in the absence of any other reagents.^20,21^ A small ratio of HPMo_12_ oxidant can perform a high efficiency for the polymerization reaction of pyrrole monomers, as shown in the following equation.^22^ After dipping HPMo_12_ anchored porous polymer film into the pyrrole aqueous solution for 40 min at room temperature, quite uniform spheres are found existing in the cavities of the patterned film even after several times of washing with water (Fig. 3a). The UV-Vis spectrum shown in Fig. S1† indicates the characteristic absorption of polypyrrole based on the reported results at *ca.* 360 and 480 nm,^23^ in contrast to no absorption appearing at those positions before the oxidation polymerization. A close examination of the film reveals that the polypyrrole particles are in tablet shape with several hundred nanometers, as shown in Fig. 3b. To further demonstrate the polypyrrole particles deriving from the oxidative polymerization of pyrrole monomer, the XPS spectra of the patterned porous film after the reaction are carried out as shown in Fig. S2.† Due to the weak interaction among P123, heteropoly acids and polypyrrole objects, a partial weight loss occurs during the washing procedure after the polymerization, which leads to a low coverage of the polypyrrole polymers in the cavities. The low coverage of the polypyrrole spheres in the cavities could be improved through the optimization of the chemical composition of the cavities by using substituted materials with a bit stronger interaction.1
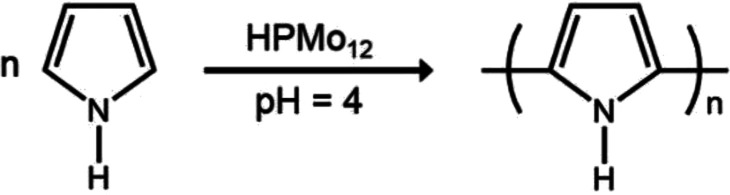


### Local reduction of silver ions in the cavities

**Fig. 4:**
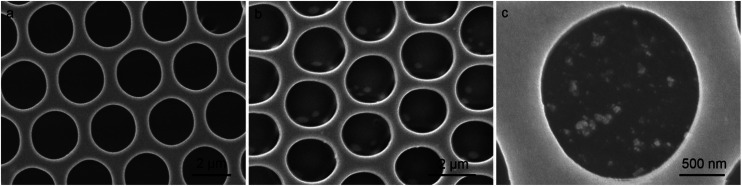
SEM images of HPW_12_/PMMA porous films (a) without and (b) with the irradiation of UV light for 15 min and then dipped in silver nitrate aqueous solution for 30 min, where (c) is the amplification of (b) in one cavity.

It is known that the heteropoly acids can be transferred to its reduced state (some metal ions change the oxidized state from M^6+^ to M^5+^) through UV light photoirradiation in solution.^24^ The blue colored heteropoly acids in its reduced state can be used for the reduction of added metal ions (from M^+^ to M^0^).^25,26^ In the present study, we employed such reaction for the *in situ* reduction of silver ions in the patterned cavities, as indicated in the eqn (S1).† After the irradiation with UV light (wavelength < 400 nm) for *ca.* 15 min, the HPW_12_ patterned film is sucked in AgNO_3_ aqueous solution for *ca.* 30 min and then washed with water. The emergence of the absorption band at *ca.* 410 nm in the UV-Vis spectrum confirms the reduction of silver ions and the formation of nanoparticles (Fig. S3†). Further, the XRD pattern also demonstrates the formation of silver nanoparticles after the treatment procedures due to the appearance of characteristic peaks for silver element (Fig. S4†). Compared with the SEM image of the virgin film (Fig. 4a), the locally dispersed Ag nanoparticles with spherical morphology in a broad size distribution indicates the successful *in situ* preparation in the cavities of the patterned film. Because the polymer film is stable in aqueous solution, the geometric morphology of the pattern cavities is well maintained (Fig. 4b and c). In addition, the EDX analysis is also an impactful method to determine the distribution of silver nanoparticles, because no silver element is contained in the original porous films. As expected, the silver element in blue color is observed locating in the domains where the silver nanoparticles stay in the cavities, as seen in Fig. S5.† To further confirm the nanoparticles deriving from the reduction of silver ions, the XPS spectra of the patterned porous film after the reaction are carried out as seen in Fig. S6.† The silver element is checked out although only a few of silver nanoparticles at the top surface of the porous film due to the washing step. Some larger particles with the dimension in several hundred nanometers are obtained because of the aggregation of small silver particles, accompanied by the prolongation of the reaction time. In comparison with the pattern directly prepared by pure organic modified gold or silver nanoparticles, the present hybrid film bearing heteropoly acids decorated cavities seems more efficient in gathering the nanoparticles in the cavities of the film.^27,28^ Moreover, a small number of silver and polypyrrole nanoparticles can be fall from the cavities on the porous film after the repetitive washing steps. However, most of silver and polypyrrole nanoparticles could be separated from the cavities after the multiple sonication and washing treatments, as seen in Fig. S7.†

## Conclusions

In conclusion, the porous films, where the heteropoly acids are assembled into the cavities and the polymer are distributed on the framework, have been developed through the microemulsion approach. The process is much simpler and more facile for fabricating chemically modified cavities on the polymer surface while the pattern formation. Herein, the heteropoly acids are locally assembled in the patterned sites on the polymer surface in one step, saving some complicated steps for the decoration of cavities which is applied in the breath figure. Interestingly, the excellent features of the heteropoly acids are maintained. Then, the simple functionalization of the modification component heteropoly acids for the reduction of metal ions and the oxidation polymerization of pyrrole in the local position proves the role of the chemical modified cavities and potential applications in material science and biomaterials. Furthermore, it can be envisioned that other aqueous-soluble molecules and nanoparticles with diverse physical, chemical or biological properties also are favorable for the functionalization of the porous polymer films, which further endow the films with a variety of potential applications in catalysis, sensor, biomedicine, and so forth.

## Conflicts of interest

There are no conflicts to declare.

## Supplementary Material

RA-008-C8RA07939K-s001
