# Supplementary material for: Assembly of heteropoly acid into localized porous structures for in situ preparation of silver and polypyrrole nanoparticles
Source: RSC Adv. 2018 Oct 30;8(64):36558–62. doi: 10.1039/c8ra07939k (PMC9088865; doi:10.1039/c8ra07939k)
Supplement: RA-008-C8RA07939K-s001 [file RA-008-C8RA07939K-s001.pdf]

Electronic Supplementary Material (ESI) for RSC Advances.
This journal is © The Royal Society of Chemistry 2018

# Supplementary Information

# Assembly of Heteropoly Acid into Localized Porous Structures for In-situ Preparation of Sliver and Polypyrrole Nanoparticles

Jing Liang*[a], Lei Yu[b], Jiangyong Zhang,[a] Shixiong Zhao[c], Jiejing Zhang[a], Jianfeng Zhang[a]

[a] College of life science, Jilin Agricultural University, Changchun 130118, China

[b] Jilin Radion and TV University, Changchun 130022, China

[c] Dalian Institute of Chemical Physics, Chinese Academy of Science, 116023, China

E-mail: liangjing@jlau.edu.cn

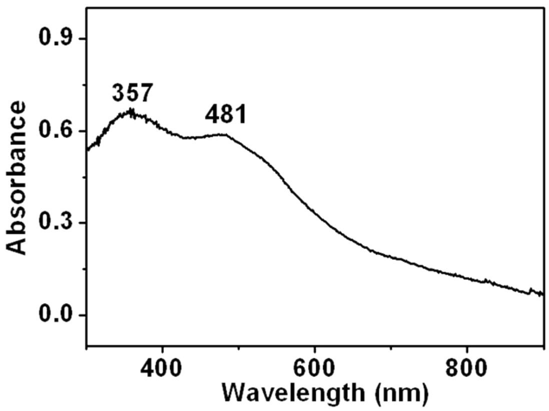


**Fig. S1** UV-Vis spectrum of $HPMo_{12}$/PMMA film on quartz substrate after the in situ polymerization (PPy spheres) prepared by dipping the hybrid porous film in 0.1 M of pyrrole aqueous solution under pH 4 for 40 min.

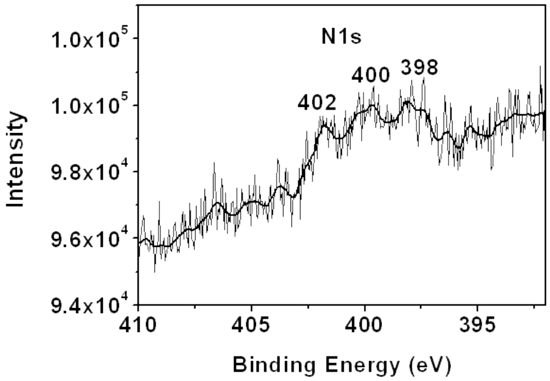

**Fig. S2** XPS spectrum of N element on the $HPMo_{12}$/PMMA film after encountering 40 min of its dipping in pH 4 of pyrrole aqueous solution.

$$POM^{n-} \xrightarrow{UV} POM^{(n-1)-}$$

$$POM^{(n-1)-} + Ag^{+} \longrightarrow POM^{n-} + Ag$$

**Eq. S1** The chemical process of reduction of silver ions by heteropoly acids.

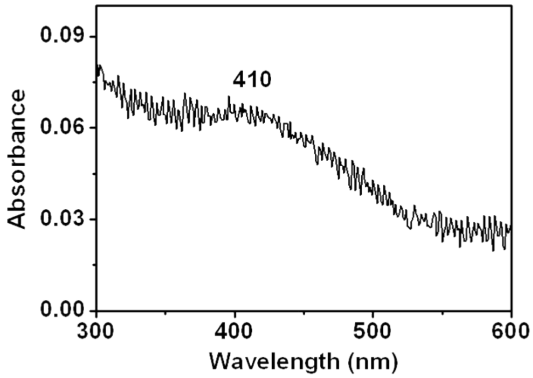


**Fig. S3** UV-vis spectrum of $HPW_{12}$/PMMA film on quartz substrate containing Ag nanoparticles prepared with the irradiation of UV light for 15 min and then dipped in silver nitrate aqueous solution for 30 min.

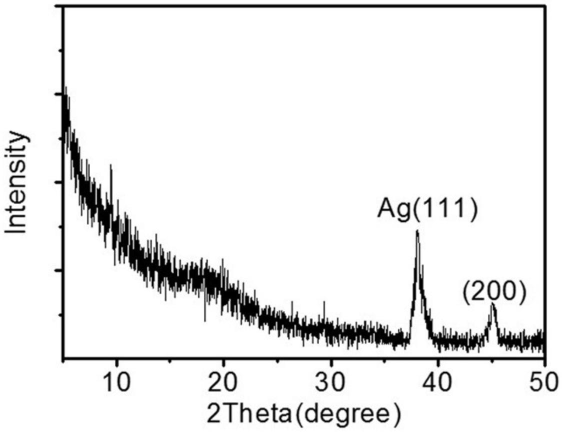


**Fig. S4** XRD spectrum of Ag nanoparticles on the $HPW_{12}$/PMMA film after 15 min of UV light irradiation for the film and then 30 min of dipping in silver nitrate aqueous solution.

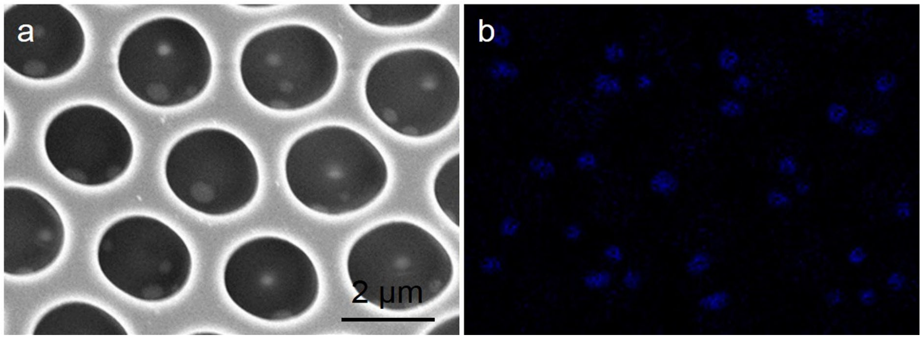


**Fig. S5** (a) SEM image and (b) EDX analysis of PMMA porous films containing silver nanoparticles, respectively.

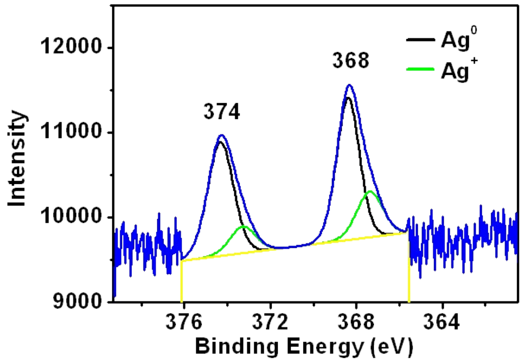


**Fig. S6** XPS spectrum of Ag element on the $HPW_{12}$/PMMA film after 15 min of UV light irradiation for the film and then 30 min of dipping in silver nitrate aqueous solution.

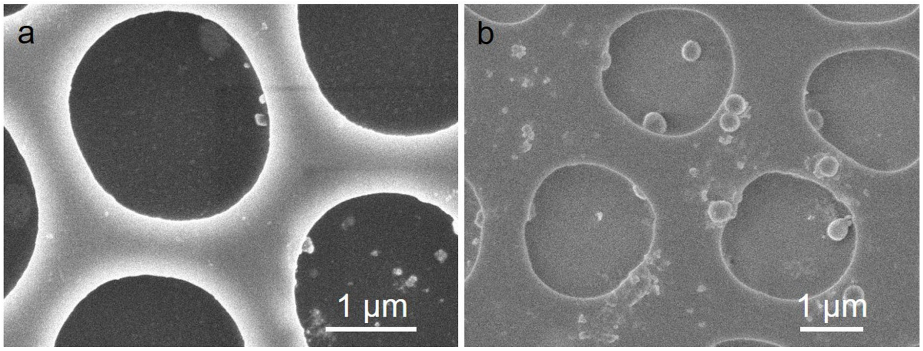


**Fig. S7** SEM images of (a) silver and (b) polypyrrole nanoparticles contained porous film with sonication and washing treatments, respectively.
